# Supplementary material for: Near-chromosomal de novo assembly of Bengal tiger genome reveals genetic hallmarks of apex predation
Source: Gigascience. 2022 Dec 28;12:giac112. doi: 10.1093/gigascience/giac112 (PMC9795480; doi:10.1093/gigascience/giac112)
Supplement: giac112_Response_to_Reviewer_Comments_Original_Submission [file giac112_response_to_reviewer_comments_original_submission.pdf]

Author's Response To Reviewer Comments

Close

Point-by-point Reviewer Response

Reviewer #1: This manuscript is about assemblies of Bengal tigers. It is a great improvement over past two tiger genome assemblies. The assemblies' quality is unprecedented (exceeding perhaps any feline genome in terms of contiguity).

>This represented a ~50x improvement in genome contiguity (see materials and methods). PanTigT.MC.v2

What was the most important factor in this big jump of improvement in length?

>> Long-read sequencing significantly increases the contiguity of genome assemblies. Further, the use of optical mapping and chromosome conformation capture-based sequencing enabled near-chromosomal genome assembly.

>the overall contiguity was better than the domestic cat reference genome

The quality comparison section is informative.

>> Thank you

>We identified the "repetitive elements" in the genome by combining both

==> repeat elements is better.

How close are the two genomes (MC & SI)?

>> We mapped the two genome assemblies with minimap2 and called variants with paftools.js (https://github.com/lh3/minimap2/blob/master/cookbook.md). Briefly, using the MC genome as the reference, we aligned SI genome and detected 2196239 SNPs per 2087495282 aligned bases. This translates to ~1.05 SNPs/kb of the SI genome. We have updated the manuscript to highlight this observation (Lines 144-146).

>> Furthermore, the populations that the two samples (MC and SI) belong to have an average Fst (fixation index score) of 0.28 (Armstrong et al. 2021, Khan et al. 2021) suggesting that the two animals belong to distinct population groups.

This reviewer finds it a great contribution to existing feline genome assemblies.
The authors have done all the usual QC and constructed really high quality assemblies.
>> Thank you.

Reviewer #2: The submitted manuscript 'Near-chromosomal de novo assembly of Bengal tiger genome reveals genetic hallmarks of apex-predation' assemble the high-quality near-chromosomal leveled reference genomes of Bengal tiger, which will be of great significance for the conservation and rejuvenation of tigers, even other endangered felids.
I have some comments on this manuscript:
1. Considering this the assembled genome used the Hic technology to figure out the chromosome structure, the figure of Hic results need to be presented. While, the assemble of sex chromosome always attract attentions, especially Y chromosome of tiger. More detailed information need to be specified, such

as the conserved Y chromosome genes compared to other mammals, or whether there are tiger-specific Y linked gene has been observed or not.
>> We have included the figure showing the contact maps as supplementary figures in the revised manuscript (Supplementary Figure 4).
>> We identified 44 protein-coding genes in the Y-linked scaffolds. All the 44 genes had a homolog in other Felidae species' Y-linked scaffolds (43/44 genes had >90% identity), suggesting a high level of conservation of Y chromosome genes in Felidae. We have now included these results in the supplementary tables (Supplementary Table 1o) and have revised our manuscript to highlight this (Lines 229-231).

2. In this work, authors used four zoo-bred individuals with known pedigree to test the inbreeding index of ROH and intend to evaluate the assembly quality. But I don't find any information about these four individuals and I guess they should be Bengal tigers. If it is the case, the question is that the quantity of ROH will not be only decided by the reference quality, but also the divergence between the target resequencing data and the used reference genome. That is to say, if the resequencing data and the reference genome are all from the same tiger sub-species, Bengal tiger, the quantity of ROH supposed to be more than that of the different sub-species comparison, which may not be an appropriate method used to evaluate the assembly quality.
>> The zoo tigers used to assess the quality of ROH are Bengal tigers. Given that the divergence among tiger subspecies is <10,000-50,000 years (Armstrong et al. 2021), a bias in estimating ROH is not expected in our analysis (Armstrong et al. 2021). As previously reported, a reference bias rendering individuals from other subspecies to be less inbred or having more diversity has thus far not been observed (Armstrong et al. 2021, Cho et al, 2013).

3. I have some advice about the evolutionary divergence calibrations. Using some other species which have closer phylogenetic relationship might be better, according to their shared similar substitution rate and generation time, for instance, other species of Panthera.
>> Our analysis used only well-annotated, high-quality Felidae genomes (including Panthera) as assessed by the BUSCO completeness. As more high-quality genomes and annotations become available, future studies can focus on revising/improving the analysis.

4. The format of references part need to be rechecked.
>> Thank you. We will work with the editorial team to format the references per journal guidelines.

Close
