# Supplementary material for: Near-chromosomal de novo assembly of Bengal tiger genome reveals genetic hallmarks of apex predation
Source: Gigascience. 2022 Dec 28;12:giac112. doi: 10.1093/gigascience/giac112 (PMC9795480; doi:10.1093/gigascience/giac112)
Supplement: giac112_Response_to_Reviewer_Comments_Revision_1 [file giac112_response_to_reviewer_comments_revision_1.pdf]

Author's Response To Reviewer Comments

Close

Reviewer reports:
Reviewer #2: The author has made the modification to the manuscript following reviewer's comments. I thought this version can met the quality requirements of GigaScience.
Here, I only have a minor suggestion. In a consanguineous communitie, it should be found more longer ROH in highly inbreeded individual than that of non-inbred individual. In this paper, four re-sequenced zoo-bred tigers (Bengal tiger) data are mapped three different tiger reference genomes to evaluate the contiguity of each genome assembly, but these four sampled Bengal tigers are closely related to the tiger individual which has been used for the genome assemble, then the reported long ROH will not be a very appropriate parameter for the comparison of different reference genomes.

>> The zoo individuals used in this study are from the Northeastern part of India (state of Orissa). Both SI and MC are wild animals (genomes reported in this paper) from southwestern and northwestern India, respectively, and are from distinct population groups (Fst = 0.28). Therefore, the patterns we observe in our study are more likely to be dictated by genome quality than the subspecies of the reference genome.

Close
