# Supplementary material for: Near-chromosomal de novo assembly of Bengal tiger genome reveals genetic hallmarks of apex predation
Source: Gigascience. 2022 Dec 28;12:giac112. doi: 10.1093/gigascience/giac112 (PMC9795480; doi:10.1093/gigascience/giac112)
Supplement: giac112_Reviewer_2_Report_Original_Submission — Gang Li -- 6/30/2022 Reviewed [file giac112_reviewer_2_report_original_submission.pdf]

Reviewer Report

Title: Near-chromosomal de novo assembly of Bengal tiger genome reveals genetic hallmarks of apex-predation

Version: Original Submission **Date:** 6/30/2022

Reviewer name: Gang Li

Reviewer Comments to Author:

The submitted manuscript 'Near-chromosomal de novo assembly of Bengal tiger genome reveals genetic hallmarks of apex-predation' assemble the high-quality near-chromosomal leveled reference genomes of Bengal tiger, which will be of great significance for the conservation and rejuvenation of tigers, even other endangered felids.I have some comments on this manuscript:1. Considering this the assembled genome used the Hic technology to figure out the chromosome structure, the figure of Hic results need to be presented. While, the assemble of sex chromosome always attract attentions, especially Y chromosome of tiger. More detailed information need to be specified, such as the conserved Y chromosome genes compared to other mammals, or whether there are tiger-specific Y linked gene has been observed or not.2. In this work, authors used four zoo-bred individuals with known pedigree to test the inbreeding index of ROH and intend to evaluate the assembly quality. But I don't find any information about these four individuals and I guess they should be Bengal tigers. If it is the case, the question is that the quantity of ROH will not be only decided by the reference quality, but also the divergence between the target resequencing date and the used reference genome. That is to say, if the resequencing data and the reference genome are all from the same tiger sub-species, Bengal tiger, the quantity of ROH supposed to be more than that of the different sub-species comparison, which may not be an appropriate method used to evaluate the assembly quality.3. I have some advice about the evolutionary divergence calibrations. Using some other species which have closer phylogenetic relationship might be better, according to their shared similar substitution rate and generation time, for instance, other species of Panthera .4. The format of references part need to be rechecked.

Level of Interest

Please indicate how interesting you found the manuscript: Choose an item.

Quality of Written English

Please indicate the quality of language in the manuscript: Choose an item.

Declaration of Competing Interests

Please complete a declaration of competing interests, considering the following questions:

- Have you in the past five years received reimbursements, fees, funding, or salary from an organisation that may in any way gain or lose financially from the publication of this manuscript, either now or in the future?
- Do you hold any stocks or shares in an organisation that may in any way gain or lose financially from the publication of this manuscript, either now or in the future?
- Do you hold or are you currently applying for any patents relating to the content of the manuscript?
- Have you received reimbursements, fees, funding, or salary from an organization that holds or has applied for patents relating to the content of the manuscript?
- Do you have any other financial competing interests?
- Do you have any non-financial competing interests in relation to this paper?

If you can answer no to all of the above, write 'I declare that I have no competing interests' below. If your reply is yes to any, please give details below.

I declare that I have no competing interests

I agree to the open peer review policy of the journal. I understand that my name will be included on my report to the authors and, if the manuscript is accepted for publication, my named report including any attachments I upload will be posted on the website along with the authors' responses. I agree for my report to be made available under an Open Access Creative Commons CC-BY license (http://creativecommons.org/licenses/by/4.0/). I understand that any comments which I do not wish to be included in my named report can be included as confidential comments to the editors, which will not be published.

Choose an item.

To further support our reviewers, we have joined with Publons, where you can gain additional credit to further highlight your hard work (see: https://publons.com/journal/530/gigascience). On publication of this paper, your review will be automatically added to Publons, you can then choose whether or not to claim your Publons credit. I understand this statement.

Yes Choose an item.
